# Diarrhea management: from pathophysiology to microbiota modulation

**DOI:** 10.1177/17562848261424324

**Published:** 2026-02-22

**Authors:** Giovanni Marasco, David Meacci, Giovanni Sarnelli, Cesare Tosetti, Cesare Cremon, Edoardo Vincenzo Savarino, Giovanni Barbara

**Affiliations:** IRCCS Azienda Ospedaliero Universitaria di Bologna, Bologna, Italy; Department of Medical and Surgical Sciences, University of Bologna, Bologna, Italy; IRCCS Azienda Ospedaliero Universitaria di Bologna, Bologna, Italy; Department of Medical and Surgical Sciences, University of Bologna, Bologna, Italy; Digestive and Nutritional Pathophysiology Unit, Department of Clinical Medicine and Surgery, University of Naples “Federico II,” Naples, Italy; Primary Care, Porretta Terme, Bologna, Italy; IRCCS Azienda Ospedaliero Universitaria di Bologna, Bologna, Italy; Gastroenterology Unit, Department of Surgery, Oncology and Gastroenterology, University of Padua, Padua, Italy; IRCCS Azienda Ospedaliero Universitaria di Bologna, Via Massarenti, 9, Bologna 40138, Italy; Department of Medical and Surgical Sciences, University of Bologna, Bologna, Italy

**Keywords:** acute diarrhea, antibiotics, chronic diarrhea, diverticular disease, dysbiosis, fecal microbiota transplantation, gut microbiota, inflammatory bowel disease, irritable bowel syndrome, prebiotics, probiotics, small intestine bacterial overgrowth

## Abstract

Diarrhea, whether acute or chronic, is a common clinical condition with numerous causes that collectively impose significant health, economic, social, and psychological burdens worldwide. Based on its duration, diarrhea is classified as acute when lasting less than 2 weeks and chronic when persisting for more than 4 weeks. From a pathophysiological standpoint, diarrhea can be categorized into four main types: osmotic, secretory, inflammatory, and motility-related. Acute diarrhea is most commonly caused by infectious gastroenteritis and tends to have a self-limited course. In contrast, chronic diarrhea presents a more complex diagnostic challenge due to its varied etiologies and clinical presentations. A shared feature among many causes of both acute and chronic diarrhea is an alteration in the gut microbiota, a condition referred to as dysbiosis. While acute infections often result in temporary microbial imbalance, chronic conditions such as irritable bowel syndrome and symptomatic uncomplicated diverticular disease are associated with persistent dysbiosis. This review aims to explore the most prevalent causes and underlying mechanisms of acute and chronic diarrhea, with a particular focus on the role of the gut microbiota. It will also examine the principal therapeutic strategies aimed at modulating intestinal microbiota, including prebiotics, probiotics, antibiotics, and fecal microbiota transplantation.

## Background

Acute diarrhea affects approximately 2.3 billion people worldwide each year and is responsible for an estimated 1400 deaths daily.^1,2^ Most of these cases occur in children under 5 years of age living in developing countries, who, despite representing only 10% of the global population, account for 40% of diarrheal deaths. Contributing factors include contaminated water sources, malnutrition, poor living conditions, and limited access to vaccines. In these settings, survivors of the acute phase remain at increased risk for persistent diarrhea, growth faltering, and cognitive impairment.^3,4^ Acute diarrhea also affects populations in high-income countries, though it rarely results in death. In the United States, it causes approximately 300 deaths annually.^5^ Nevertheless, it remains one of the most frequent reasons for primary care visits, accounting for about 1.5 million outpatient visits and approximately 220,000 hospital admissions each year. In developed countries, acute gastroenteritis leads to hospitalization in about 8–15 per 1000 cases, with an estimated annual healthcare cost of $23 million.^6,7^ Importantly, acute diarrhea can result in both short- and long-term complications. Bacterial gastroenteritis has been linked to the development of reactive arthritis, hemolytic uremic syndrome, Guillain–Barré syndrome, and a heightened risk of inflammatory bowel disease (IBD).^8,9^ Moreover, acute gastroenteritis is a well-recognized risk factor for the development of chronic gastrointestinal disorders, particularly disorders of gut-brain interaction (DGBI).^10^ Among the most common post-infectious DGBIs are functional dyspepsia^11^ and irritable bowel syndrome (IBS).^12^ IBS is a common cause of chronic diarrhea and one of the most frequent reasons for referral to a gastroenterologist.^13^ Although its exact prevalence is difficult to determine, chronic diarrhea is estimated to affect between 4% and 14% of the general population.^14^ The economic burden is considerable; recent data show that patients with chronic diarrhea incur healthcare costs exceeding $8000 per year, including consultations and prescriptions, compared to control subjects.^15^ Beyond financial implications, chronic diarrhea significantly impairs health-related quality of life. It is associated with increased absenteeism, reduced work productivity, and higher rates of psychological comorbidities.^16^ In conclusion, persistent abdominal symptoms and the often-limited availability of effective treatments contribute to elevated levels of anxiety and depression in affected individuals compared to healthy controls.^17^ This review aims to explore the most prevalent causes and underlying mechanisms of acute and chronic diarrhea, with a particular focus on the role of the gut microbiota in chronic diarrhea and its modulation. We performed a critical evaluation of the available literature addressing the principal causes of acute and chronic diarrhea, as well as the involvement of the gut microbiota in their pathogenesis, including studies published up to July 2025, selecting only articles in English. Articles were searched using the following keywords: “acute diarrhea” OR “chronic diarrhea” AND “gut microbiota.” Two authors performed study selection (G.M. and D.M.), while a third author (G.B.) resolved any disagreement for the inclusion of studies.

### Definition and classification

The daily amount of water reaching the colon is approximately 1.5 L, yet only about 200 mL is typically expelled with stools.^18^ This value is influenced by an individual’s dietary habits; for instance, the Mediterranean diet, which is rich in fiber, may result in stool output exceeding 300 mL/day. Other variables that influence stool daily output are age, sex, and body weight.^19^

The variability in normal water content in stools complicates the precise definition of diarrhea, which has historically been described as stool output exceeding 200 g/day.^20^ Alternatively, some authors define diarrhea based on stool frequency, considering more than 3 evacuations per day as indicative of the condition.^21^ However, the defining characteristic of diarrhea is more closely related to stool consistency. An increased proportion of free water in stools leads to liquid evacuations, which can be readily assessed using the Bristol Stool Chart. Specifically, frequent and abnormal stools classified above type 5 are associated with diarrheal bowel movements.^22^

Consensus is lacking not only regarding diarrhea definition, but also about its classification based on duration. Most authors categorize diarrhea as acute when symptoms persist for less than 2 weeks, persistent when lasting between 2 and 4 weeks, and chronic when diarrheal episodes extend beyond 4 weeks.^23,24^

## Clinical features

### Acute diarrhea

**Figure 1.**
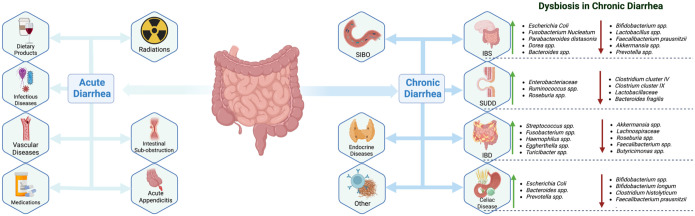
Main causes of acute and chronic diarrhea with a focus on chronic dysbiosis.

Acute and chronic diarrhea have distinct etiologies (Figure 1). Acute diarrhea is primarily caused by infectious agents, with *Norovirus* being the most common pathogen, accounting for approximately 50% of all diarrhea cases in Western populations.^25^ Other viral agents responsible for diarrhea outbreaks include *Rotavirus*, *Astrovirus*, and *Adenovirus*.^26^

Bacterial agents are the leading cause of traveler’s diarrhea: Enterotoxic and Shiga toxin-producing *Escherichia coli*, *Bacillus cereus*, *Shigella dysenteriae*, *Clostridioides difficile*, *Vibrio cholerae* produce toxins that induce either watery or bloody diarrhea.^27^ In addition, bacterial genera such as *Salmonella*, *Yersinia*, and *Campylobacter* can invade the intestinal mucosa, impairing absorption and leading to watery or bloodystools.^28^ Protozoa infections may also cause acute diarrhea in both immunocompetent and immunocompromised individuals: *Giardia lamblia* and *Entamoeba histolytica*, common waterborne contaminants, can induce watery diarrhea in healthy individuals, whereas *Cryptosporidium parvum* is a frequent cause of diarrhea in patients with AIDS or other immunosuppressive conditions.^29^

Noninfectious causes of acute diarrhea include exposure to radiation therapy and certain medications, such as antibiotics or chemotherapy, which can cause diarrhea as an adverse event.^30,31^ Vascular diseases affecting the gastrointestinal tract, including ischemic colitis and mesenteric ischemia, may present with symptoms such as abdominal pain, hematochezia, and diarrhea.^32,33^ Furthermore, some conditions requiring surgical treatment, such as appendicitis or mechanical intestinal sub-obstruction, can manifest with an episode of acute diarrhea.^34,35^ Finally, acute diarrhea may not always be linked to pathological conditions but can instead result from dietary factors that stimulate gastrointestinal motility, such as alcohol or caffeine consumption.^28^

### Chronic diarrhea

While acute gastroenteritis is the primary etiological factor in most cases of acute diarrhea, identifying the underlying cause of chronic diarrhea is often more complex and challenging. Chronic diarrhea can present with watery, fatty (steatorrhea), or bloody diarrhea, and stool characteristics serve as a valuable diagnostic clue in determining its origin.

Watery diarrhea is the most nonspecific presentation, as numerous conditions can result in increased stool water content, particularly in the early stages of the disease. Persistent watery diarrhea is commonly associated with Crohn disease,^36^ IBS,^37^ microscopic colitis,^38^ small intestinal bowel overgrowth (SIBO),^39^ Symptomatic Uncomplicated Diverticular Disease (SUDD),^40^ autonomic neuropathy, drugs, hormone-secreting tumors (e.g., gastrinoma, VIPoma, and carcinoid tumors), thyroid and parathyroid disorders.^22^

Steatorrhea indicates an underlying malabsorption or maldigestion disorder. Malabsorption is typically due to small bowel villous alteration, such as in coeliac disease,^41^ Giardiasis, Whipple’s disease, enteric lymphoma, amyloidosis, SIBO, and HIV enteropathy.^22^ Conversely, maldigestion may result from lactase deficiency or pancreatic insufficiency, often secondary to chronic pancreatitis or pancreatic tumors.^42^ Altered bile acid metabolism and delivery, due to cholestatic liver disease such as primary biliary cholangitis, primary sclerosing cholangitis,^43^ or reduced biliary acid pool following distal ileal resection or Crohn’s disease involving the distal ileum, can also contribute to steatorrhea.^44^ Since steatorrhea typically reflects advanced malabsorption disease, it’s often preceded by watery diarrhea.

Chronic bloody diarrhea is indicative of significant colonic inflammation, which may be caused by chronic infections (e.g., *C. difficile* colitis or cytomegalovirus (CMV) colitis),^45^ autoinflammatory disease (most notably ulcerative colitis, and less frequently, Crohn disease),^36^ and gastrointestinal vascular disorders such as ischemic colitis.^32^

## Physiopathology

Diarrhea results from an imbalance between absorption, secretion, and gut motility within the intestinal tract. Based on the underlying pathophysiological mechanisms, it can be classified into osmotic, secretory, inflammatory, and motility-related. However, despite this classification, diarrhea often arises from multiple mechanisms acting simultaneously.

### Osmotic diarrhea

Osmotic diarrhea occurs when an excess of osmotically active substances in the intestinal lumen draws water from the abluminal to the adluminal side, exceeding the absorbative capacity of the gastrointestinal tract. A key characteristic of this type of diarrhea is its reduction or resolution during periods of fasting. Osmotic diarrhea is commonly associated with maldigestion and malabsorption disorders. Undigested particles in the colonic lumen retain water molecules, leading to increased stool frequency and reduced consistency. For example, pancreatic insufficiency results in a decreased secretion of metabolically active digestive enzymes, preventing proper food digestion and causing water retention in the intestinal lumen, ultimately leading to diarrhea.^46^ In addition, a reduction in the absorptive surface of the small intestine or a damaged villous system—such as in coeliac disease or SIBO—can disrupt the balance between absorption and secretion. In these conditions, osmotically active substances reach the colon, increasing luminal water content and resulting in watery or fatty diarrhea.^47,48^

### Secretory diarrhea

Secretory diarrhea occurs when intestinal secretions exceed the absorptive capacity of the colonic tract. Gastrointestinal secretory activity can be enhanced by the overproduction of endogenous peptides. For instance, VIPoma, a neuroendocrine tumor that secretes vasoactive intestinal peptide, stimulates chloride secretion into the intestinal lumen via CFTR channels through cyclic Adenosine-Monophosphate (cAMP)-dependent mechanisms. This leads to watery diarrhea, hypokalemia, and metabolic acidosis.^49^ Several microorganisms also induce excessive intestinal secretion by producing toxins that activate ion channels. *Vibrio cholerae* secretes a toxin that increases cAMP levels, thereby stimulating chloride secretion into the lumen. To maintain the ionic balance, sodium ions (Na+) are also secreted, creating an osmotic gradient that drives water into the intestinal lumen, resulting in profuse diarrhea.^50^ *Enterotoxic E. coli* induces diarrhea through a similar mechanism.^51^

### Inflammatory diarrhea

Inflammatory diarrhea results from inflammation of the intestinal mucosa and is driven by multiple pathological mechanisms. Damage to the epithelial barrier impairs its absorptive capacity, leading to malabsorption. In addition, inflammation promotes fluid secretion into the intestinal lumen, while cellular debris from destroyed epithelial cells exhibits osmotic activity, further contributing to water retention and diarrhea.^52^ Clinically, inflammatory diarrhea is often characterized by the presence of mucus and blood in the stool, accompanied by abdominal pain and tenesmus. Laboratory findings may reveal elevated levels of leucocyte-derived proteins, such as fecal calprotectin, as well as increased serum inflammatory markers, including C-reactive protein (CRP). The most common causes of inflammatory diarrhea include Crohn’s disease and ulcerative colitis. However, certain infections, including *C. difficile*, *E. histolytica*, and CMV, can also provoke a similar inflammatory response, leading to inflammatory diarrhea.^53^

### Motor diarrhea

The absorption of water and electrolytes requires an intact mucosal surface and a sufficient contact time between luminal fluids and the intestinal epithelium. Intestinal motility plays a crucial role in mixing luminal contents, enhancing their exposure to the absorptive surface, and ensuring the proper propulsion of fecal matter in the aboral direction. While reduced gut motility can lead to constipation, excessive intestinal propulsion results in motor diarrhea. This type of diarrhea arises from a shortened contact time between luminal contents and the intestinal mucosa, impairing fluid reabsorption and leading to excessive water loss.^54^ A classic example of motor diarrhea is diabetic neuropathy, where loss of adrenergic tone in the intestinal and colonic innervation can lead to hyperperistalsis and subsequent diarrhea.^55^ Similarly, in diarrhea-predominant irritable bowel syndrome (IBS-D), exaggerated intestinal motility and an increased frequency of high-amplitude propagated contractions have been described.^56,57^

## Diagnostic algorithm

In general, acute diarrhea is a self-limited condition that usually does not require specific diagnostic evaluation, and management is often based on dietary recommendations, adequate fluid intake, and, when appropriate, probiotic administration.^28,58^ However, further evaluation is mandatory in case of alarm features, including important dehydration, high fever, bloody stools, severe abdominal pain, hypotension, or in fragile patients.^28^

In all these cases, a thorough medical history should be performed, looking for recent travel to areas at high risk for traveler’s diarrhea, the quality and type of foods recently consumed, and the use of medications.^59^ Moreover, laboratory evaluations, including complete blood count, electrolyte levels, CRP, and renal and liver function tests, might be performed to evaluate disease severity and to identify potential complications.^28^ Furthermore, stool samples should be collected to measure fecal calprotectin and to perform microscopic and culture analyses, as well as *C. difficile* toxin testing, especially in at-risk patients with recent antibiotic exposure.^28^

Imaging studies may be useful in selected cases. A plain abdominal radiograph can help to exclude an obstructive cause, which might lead to pseudo-diarrhea,^60^ while abdominal computed tomography angiography could be considered in patients with bloody diarrhea and high cardiovascular risk to rule out ischemic colitis.^61^ Moreover, contrast-enhanced CT imaging may be used to identify complications or alternative diagnoses requiring surgical management, such as acute appendicitis.^62^ Conclusively, in patients with persistent symptoms or a high pre-test probability of organic disease, endoscopic evaluation with colonoscopy or sigmoidoscopy might be performed to evaluate macroscopic findings and to collect biopsies for histological analysis, which is essential to exclude conditions presenting with acute colitis, including IBD or CMV colitis.^63^

Conversely, chronic diarrhea represents a more challenging and clinically relevant condition because of its frequent association with quality-of-life impairment and since chronic diarrhea might be a potential expression of an underlying disorder requiring targeted intervention. The diagnostic assessment to perform in case of chronic diarrhea often integrates that of the acute form and considers a wider range of etiologies. Blood tests might also include celiac serology as well as total IgA levels, evaluation of endocrine function, nutritional status analysis, including fat-soluble vitamins, as well as vitamin B9 and vitamin B12 levels.^37,64^ In patients with evidence of malabsorption or a history of pancreatic disease, fecal elastase testing may be useful to exclude exocrine pancreatic insufficiency, even if a false-positive result could be obtained in case of watery diarrhea.^65^

However, stool investigations, in selected cases, might include fecal calprotectin measurement, microscopic examination, and stool cultures. Testing for *G. lamblia* and *E. histolytica* should be considered in patients with significant risk factors, along with *C. difficile* toxin detection.^66^

Attention should be paid to immunocompromised and hematological patients, in whom chronic diarrhea might be caused by both atypical infectious pathogens (such as *Strongyloides stercoralis*, *Mycobacterium avium complex*, or CMV) and noninfectious conditions, including immune-mediated enteropathies, intestinal lymphoma, or graft-versus-host disease.^67,68^ Imaging studies, such as abdominal ultrasound, bowel ultrasound, or entero-CT, might help identify disorders of parenchymal organs, such as hepatobiliary or pancreatic diseases, as well as inflammatory conditions of the pathologic conditions involving small bowel or colon (Crohn’s disease or ulcerative colitis).^69,70^

In case of alarm features, it is mandatory to perform a colonoscopy before diagnosing IBS or SUDD. Potential red flags include a family history of colorectal cancer, celiac disease, or IBD, symptom onset after the age of 50 years, unintentional weight loss, rectal bleeding or anemia, abdominal mass, and nocturnal symptoms.^37,66^ Upper or lower endoscopic evaluation with biopsy sampling might be useful to exclude conditions such as IBD, microscopic colitis, Whipple’s disease, and immune-mediated enteropathies.^38,64,71,72^ Additional investigations, including lactose or glucose breath tests, might be used to exclude lactose intolerance or SIBO. However, due to their limited diagnostic accuracy and high false-positive rates, these tests should be used only in selected cases and interpreted with caution.^37,66^

Finally, in patients with persistent symptoms despite negative first-line investigations and with a clinical picture suggestive of bile acid diarrhea, such as postprandial or early morning diarrhea, or a history of cholecystectomy, this condition may be assessed using the ^75^Se-homocholic acid taurine test.^37,66^

For the specific diagnosis of IBS, a positive symptom-based approach with limited testing should be used, according to the Rome Criteria embraced by several guidelines.^66,73^ To date, no definitive criteria are available for the diagnosis of SUDD, although the presence of short-lasting abdominal pain in the lower quadrants is often used to define this condition when diverticula are present.^74,75^

## The role of microbiota in chronic diarrhea

The gut microbiota plays a pivotal role in maintaining intestinal health and homeostasis, and its dysregulation has been implicated in various pathological conditions, including IBD and celiac disease.^76–78^ As previously mentioned, dysbiosis—an imbalance in the composition of gut microbiota—can contribute to chronic diarrhea. Microorganisms can trigger all the previously described subtypes of diarrhea. Dysbiosis seems secondary and transient in acute diarrhea, while mainly secondary to inflammation in IBD, cytotoxic, and ischemic etiologies. However, a more central pathophysiological role for microbiota imbalances has been reported in patients with non-inflammatory chronic diarrhea, such as IBS and SUDD-associated diarrhea.

### Gut microbiota in IBS-D

IBS is a common disorder classified among the DGBI, with multifactorial pathophysiology involving immune dysregulation, visceral hypersensitivity, and altered permeability.^79,80^ Notably, the gut microbiota plays a crucial role in modulating these processes, and dysbiosis is considered a key factor in IBS pathogenesis.^81–83^ For instance, one of the most significant risk factors currently recognized for the development of IBS is a prior gastrointestinal infection, which may lead to the condition known as post-infectious IBS.^84,85^ IBS is characterized by chronic abdominal pain and alterations in bowel habits, which define its different subtypes.^37^ People with IBS-D exhibit distinct microbiota signatures compared to healthy controls and other IBS subtypes.

Several studies have investigated the microbial profile of IBS-D patients, yielding heterogeneous and sometimes conflicting results.^86^ However, a consistent finding is that IBS-D patients generally exhibit a reduced α-diversity compared to healthy subjects or to other IBS subtypes.^87,88^

A commonly reported finding regarding the IBS-D microbiota is an increased *Firmicutes*/*Bacteroidetes* ratio.^89^ Potentially harmful bacteria within the *Firmicutes* phylum, such as *Clostridia*, are frequently reported as more abundant in IBS-D patients. These bacteria can produce various toxins and proinflammatory molecules, exacerbating immune response and contributing to diarrhea and abdominal pain.^90^ Conversely, other short-chain fatty acids (SCFAs) producers, such as *Faecalibacterium prausnitzii*, which plays a pivotal role in gut homeostasis due to its anti-inflammatory properties, are significantly reduced in IBS-D patients compared to healthy controls.^91,92^

Among the various phyla, *Actinobacteria*, in particular the *Bifidobacterium* genus, are significantly reduced in both fecal and mucosal samples from IBS-D.^90^ *Bifidobacterium* strains are common colonizers of the human gut, contributing to intestinal homeostasis, immune regulation, and bile salts metabolism.^93^ Along with *Lactobacillus*—another genus commonly depleted in IBS patients^94^—*Bifidobacterium* produces bacteriocins and other antimicrobial compounds that help to prevent the colonization of pathogenic bacteria.^95,96^ Furthermore, both genera are major producers of SCFAs, including acetate, butyrate, and propionate, which represent essential nutrients for colonocytes, support intestinal barrier integrity, and exert anti-inflammatory effects.^97^ Given their crucial role in gut health, alterations in the abundance of these beneficial bacteria are likely to contribute to IBS pathophysiology.

Regarding *Proteobacteria*, IBS patients exhibit a higher abundance of this phylum compared to healthy subjects. *Enterobacteriaceae*, especially *E. coli*, show increased concentrations in both mucosal and fecal microbiota of IBS-D patients.^90,98^ *Escherichia coli* may contribute to diarrhea through multiple mechanisms, including toxin production and the release of lipopolysaccharide, which activate toll-like receptors (TLRs), promote mucosal inflammation, and increase intestinal permeability.^90^ In addition, other potentially harmful bacteria found in higher concentrations in IBS-D patients’ fecal samples include *Fusobacterium nucleatum*, *Parabacteroides distasonis*, *Lachnospiraceae*, *Bacteroides*, and *Dorea*. In contrast, a significant reduction has been observed in *Tannerella*, *Alistipes*, *Akkermansia*, and *Prevotella* compared to healthy subjects.^87,99–101^

### Gut microbiota in SUDD

SUDD is a common cause of abdominal pain and chronic diarrhea, particularly in the elderly population. SUDD represents a distinct clinical entity, separate from IBS, diverticulosis, and acute diverticulitis. Although its pathophysiology remains incompletely understood, it is thought to involve low-grade inflammation, abdominal colonic motility, and neuroimmune interactions.^102,103^ Similarly to IBS, recent evidence suggests that gut microbiota alterations may play a contributory role in the onset and progression of SUDD.^40^ To date, only a limited number of studies have investigated microbial alterations in SUDD patients, and their findings are often inconsistent.

Barbara et al.^104^ reported that SUDD patients exhibit a decreased abundance of beneficial bacteria in fecal samples. Specifically, members of *Clostridium* cluster IV, including *Faecalibacterium prausnitzii*, were significantly reduced in the fecal microbiota of both SUDD and diverticulosis patients compared to healthy controls. SUDD patients exhibit a distinct fecal microbiota composition, characterized by a reduction in *Clostridium cluster IX* and *Lactobacillaceae*, both of which are capable of producing anti-inflammatory compounds. Notably, SUDD is associated with a low-grade inflammation, primarily mediated by macrophage activation. The depletion of immunomodulatory bacterial species may disrupt the balance between pro- and anti-inflammatory factors, potentially exacerbating the inflammatory response. Furthermore, studies have demonstrated an increased abundance of *Enterobacteriaceae* and a decreased richness of *Bacteroides*/*Prevotella* and *Akkermansia* in the colonic segment affected by diverticula, compared to the distant unaffected sites.

Other studies have reported different results. Tursi et al.^105^ observed a higher abundance of *Akkermansia muciniphila* in fecal samples of SUDD patients compared to healthy subjects. However, since *Akkermansia* primarily resides in the mucous layer, its increased presence in fecal samples may reflect an underlying imbalance in mucus production and microbial homeostasis.^106^

Alteration in microbial diversity has also been linked to specific SUDD symptoms. For instance, bloating has been positively associated with *Ruminococcus* abundance and inversely correlated with *Roseburia* richness, while elevated *Cyanobacterium* levels were directly associated with higher pain scores.^107^

In conclusion, diverticular disease has been associated with a higher abundance of *Enterobacteriaceae* in mucosal samples^108^ and decreased presence of *Bacteroides fragilis*, *Collinsella aerofaciens*, and *C. stercoris* in stool samples compared to healthy controls.^109^

## Microbiota modulation

**Figure 2.**
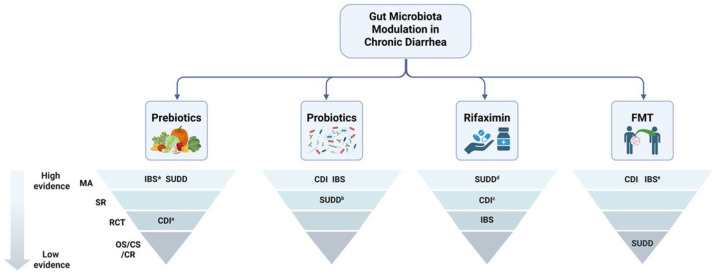
Gut microbiota modulation-based therapeutic approaches for chronic diarrhea, with the current level of evidence in literature. *Multiple meta-analyses led to conflicting results. ^a^Available only studies in association with probiotics. ^b^The only systematic review available was inconclusive in demonstrating the effectiveness of the treatment. ^c^The systematic review included only two RCTs. ^d^The meta-analysis included only four studies. ^e^Different meta-analysis led to conflicting results. CDI, *Clostridioides difficile* infection; CR, case report; CS, case series; FMT, fecal microbiota transplant; IBS, irritable bowel syndrome; MA, meta-analysis; OS, observational studies; RCT, randomized controlled trials; SR, systematic review; SUDD, symptomatic uncomplicated diverticular disease.

Dysbiosis has a crucial role in many causes of chronic diarrhea, making microbiota modulation a key therapeutic strategy for managing this condition (Figure 2). In the treatment of IBS and SUDD, various approaches aim to reshape the gut microbiota by increasing the abundance of beneficial bacteria while reducing potentially harmful species. The most studied approaches are prebiotics supplementation, probiotics intake, antibiotics administration, and fecal microbiota transplantation (FMT).

### Prebiotics

Prebiotics are substrates selectively utilized by the intestinal microbiota that confer beneficial effects to the host.^110^ Among the most well-known and widely used prebiotics are soluble dietary fibers such as fructo-oligosaccharides (FOS), galacto-oligosaccharides (GOS),^111^ and psyllium fibers.^112^ In diarrheal conditions, soluble fibers may provide therapeutic benefits not only due to their water-holding viscosity, which helps reduce stool liquidity, but also by promoting the production of SCFAs and enhancing the abundance of beneficial taxa such as *Bifidobacteria.*^113,114^

The impact of fiber supplementation in IBS patients has shown mixed results. A meta-analysis involving 15 randomized controlled trials (RCTs) and 946 patients found that soluble fiber—particularly psyllium—was effective in reducing IBS symptoms compared to placebo, although a higher incidence of adverse events was observed in the fiber-treated groups.^115^ Conversely, another meta-analysis focusing on prebiotics DGBI failed to demonstrate a significant improvement in IBS symptoms. Notably, patients treated with FOS experienced a worsening of fermentation-related symptoms such as bloating, despite showing increased fecal *Bifidobacteria* levels compared to placebo.^116^

Fiber intake has also been investigated in the context of SUDD treatment and prevention. High dietary fiber consumption has been associated with a 41% reduced risk of developing diverticular disease compared to low fiber intake.^117^ A meta-analysis by Eberhardt et al.^118^ reported that fiber supplementation (42 g/day) significantly increased stool weight (*p* < 0.0001), but had no significant impact on gastrointestinal symptoms or stool transit time. However, another recent meta-analysis indicated that the consumption of fruit and cereal fibers was linked to a reduced risk of diverticulitis and related hospitalizations.^119^

### Probiotics

The World Health Organization defines probiotics as “live microorganisms which, when administered in adequate amounts, confer a health benefit on the host.”^120,121^

The World Gastroenterology Organization Practice Guideline on probiotics and prebiotics endorses their use for several clinical indications, including the prevention of hepatic encephalopathy, the management of acute diarrhea, pouchitis, and the treatment of IBS.^58^

Several trials have evaluated the efficacy of probiotic supplementation in IBS management; however, most of the studies were affected by significant methodological limitations, including short intervention periods, small sample sizes, heterogeneity in probiotic strains and dosages, and variability in administration regimens. Furthermore, the effects of probiotics on overall gut microbiota composition have frequently been transient and not sustained over time, as reported by trials investigating the effect of probiotics in IBS patients with concurrent major depressive disorders.^122^

Although evidence on the use of probiotics in the management of IBS-D remains heterogeneous and often contradictory, and no specific strain or multistrain has been universally recommended,^123^ several guidelines support their use in IBS treatment.^41,66,124^

European guidelines generally support the use of probiotics as a group to alleviate symptoms in patients with IBS. For example, the recently published Italian guidelines for IBS management recommend probiotics for improving overall symptoms and abdominal pain. However, due to the heterogeneity of existing studies, these guidelines do not specify which strains, formulations, or combinations should be used.^37^ Similarly, the European guidelines for functional bowel disorders with diarrhea endorse the general use of probiotics in IBS and suggest that certain strains may help improve diarrhea symptoms. Nonetheless, due to limited evidence, these guidelines do not recommend probiotics for patients with functional diarrhea.^66^

Further support comes from the British guidelines, which state that probiotics may be a useful therapeutic option for managing global IBS symptoms and abdominal pain. These guidelines advise continuing probiotic use for at least 3 months before considering discontinuation, in alignment with the duration of most clinical trials (typically 12 weeks).^124^ In contrast, the American guidelines on the use of probiotics for gastrointestinal disorders are more conservative. They recommend probiotic use in IBS patients only within the context of clinical trials, citing insufficient high-quality, consistent data to support routine clinical use in this population.^125^

A recent meta-analysis involving 82 trials and over 10,000 patients demonstrated that certain probiotic combinations and specific strains may be effective in managing IBS symptoms. Overall, probiotic supplementation was found to be significantly more effective than placebo in reducing global IBS symptoms, abdominal pain, and bloating. Specifically, in patients with IBS-D, combinations with strains of *Lactobacillus* and *Bifidobacterium* showed superior efficacy compared to placebo in alleviating global IBS symptoms. However, when focusing solely on abdominal pain, only *Lactobacillus* spp. showed a statistically significant benefit in reducing pain scores.^126^ IBS is a complex, multifactorial condition influenced by various pathophysiological mechanisms, which can vary in significance from one patient to another. Therefore, a personalized treatment approach—targeting the predominant altered mechanisms in each individual—may yield superior therapeutic outcomes. For example, probiotic supplementation may be particularly effective in patients with a prominent dysbiotic component. The PROBE-IBS/1^127^ and PROBE-IBS/2^128^ trials assessed the effects of *Lactobacillus paracasei* DG on IBS symptom relief. Although a greater proportion of patients in the probiotic group reported symptom improvement compared to placebo, the difference was not statistically significant (*p* = 0.336). However, post hoc analysis of baseline fecal samples revealed that responders had a significantly higher abundance of specific bacterial taxa, particularly *C. aerofaciens*, compared to non-responders (*p* = 0.018) and healthy controls. Furthermore, probiotic administration in responders led to a notable reduction in several altered bacterial species, especially *C. aerofaciens*, a hydrogen-producing bacterium associated with accelerated colonic transit. These findings suggest that *L. paracasei* DG may be more effective in IBS patients with elevated fecal levels of *C. aerofaciens*, supporting the concept of a tailored probiotic approach in IBS management.

While the role of probiotics in IBS treatment remains uncertain, their effectiveness in managing SUDD is even more debated. As previously mentioned, SUDD pathogenesis involves an imbalance in microbiota composition and richness. Probiotic administration may help mitigate this imbalance by inhibiting proliferation through competitive mechanisms, reducing low-grade inflammation, and improving the gut barrier.^129^ A pilot RCT by Kvasnovsky et al. investigated the effects of a multistrain probiotic containing *Lactobacillus acidophilus*, *Lactobacillus casei*, and *Enterococcus faecium* on SUDD symptoms. While no significant primary outcomes were achieved, the probiotic group showed notable improvements in secondary endpoints, including a reduction in diarrhea, constipation, and mucorrhea, compared to placebo.^130^ Additionally, other studies have shown that a combination of *L. casei* and mesalazine was more effective than placebo in maintaining remission SUDD patients. Lahner et al. explored the role of *L. paracasei* combined with a high-fiber diet in SUDD management over 6 months. Compared to baseline, the symbiotic-treated group experienced a significant reduction in the proportion of patients reporting abdominal pain after 3 and 6 months (100% vs 35% vs 25%, respectively, *p* < 0.001). Furthermore, a significant reduction in abdominal bloating was observed after 3 months and remained stable at 6 months (95% vs 60% at 3 months, *p* < 0.005).^131^ Beyond symptom relief, probiotic intake in SUDD has also been linked to recurrence prevention. A combination of mesalazine and *L. casei CNCM I-1572*, administered for 10 days/month over 12 months, proved superior to placebo in preventing symptom recurrence in SUDD patients, particularly when used together.^132^

These studies suggest a beneficial role for probiotics in SUDD management. In fact, the 2019 international consensus on diverticulosis and diverticular disease acknowledged that there is evidence supporting the use of probiotics to alleviate symptoms in SUDD patients.^133^ However, due to the variability and limited quality of the available studies, a systematic review was unable to conclusively demonstrate the effectiveness of probiotics in SUDD treatment.^134^

### Antibiotics

In the past, various antibiotics have been evaluated for IBS treatment, aiming to target the microbial populations that are overrepresented in these patients. Some studies assessed neomycin^135^ and norfloxacin^136^ efficacy in alleviating IBS symptoms. While these antibiotics achieved favorable response rates in the treated group (35.0% and 37.5%, respectively), compared to placebo (11% and 0%), their use is not recommended due to the risk of various adverse effects, such as ototoxicity or neuropathies.^37^

The only antibiotic currently recommended for the treatment of IBS without constipation is rifaximin-α, first synthesized in 1982 from rifamycin.^137^ Rifaximin-α is a non-absorbable oral antibiotic with a broad-spectrum activity against both aerobic and anaerobic bacteria, including Gram-positive and Gram-negative species.^138,139^ It is often referred to as an “eubiotic” due to its microbiota-modulating properties.^140^ Rifaximin-α treatment has been associated with an increased abundance of beneficial bacteria species, such as *Lactobacillus* and *Bifidobacterium*, alongside a reduction of harmful microbes such as members of *Peptostreptococcaceae* and *Enterobacteriaceae* taxa.^139^ Additionally, rifaximin showed anti-inflammatory properties, by binding to the Pregnane X Receptor, which regulates inflammation, and by reducing abdominal pain through modulation of transient receptor potential vanilloid 1 channels (TRPV1).^141,142^

The main studies reporting on the efficacy of rifaximin in gastrointestinal disease with chronic diarrhea are summarized in Table 1. The strongest evidence supporting rifaximin’s benefits in IBS-D treatment comes from two large-scale studies, TARGET 1 and TARGET 2, which involved over 1000 patients. In both studies, a 14-day course of rifaximin daily intake was associated with a significant reduction of global IBS symptoms (40.7% vs 31.7%, *p* < 0.001), bloating (40.2% vs 30.3%, *p* < 0.001), and stool consistency improvement during treatment and for up to 4 weeks post-treatment.^143^ However, since many patients experienced symptom relapse after a few weeks, the TARGET 3 trial evaluated the efficacy and the safety of retreatment with rifaximin. Patients who received an additional course of rifaximin showed a significantly higher rate of improvement in abdominal pain and stool consistency, compared to placebo (38.1% vs 31.5%, *p* = 0.03).^144^ Fodor et al.^145^ evaluated the impact of rifaximin on gut microbiota composition in patients from the TARGET 3 trial. Using 16S rRNA gene sequencing, they compared fecal microbiota before and after rifaximin retreatment and observed a modest reduction in several potentially harmful taxa, including *Enterococcaceae*, *Peptostreptococcaceae*, and *Enterobacteriaceae*. However, the effect on microbial composition was limited and transient—by 4 weeks after the end of treatment, no significant differences were found in stool microbiota compared to baseline. These evidence reinforce rifaximin’s role in IBS-D as a first-line therapy for IBS as an effective option for symptom recurrence, with effects on microbial modulations limited in time.

**Table 1.** Main studies reporting the efficacy of rifaximin in gastrointestinal diseases with chronic diarrhea (IBS and SUDD).

Author and design	Study population	Intervention	Findings	Main limitations
IBS				
Piementel M et al., 2006; RCT	87 IBS patients	Rifaximin 400 mg three times daily for 10 days	A significantly higher proportion of patients treated with rifaximin experienced improvement in global IBS symptoms and abdominal bloating	Small sample size Only short-term data available
Lembo A et al., 2008; RCT	388 IBS patients	Rifaximin 550 mg three times daily for 2 weeks	A higher proportion of patients in the rifaximin group experienced an improvement in global IBS symptoms and abdominal bloating	Small sample size Only short-term data available
Pimentel M et al., 2011; RCT	1260 IBS patients	Rifaximin 550 mg three times daily for 2 weeks	A higher proportion of patients in the rifaximin group experienced a reduction in abdominal pain, bloating, and watery or loose stools during the first 4 weeks after treatment	Only short-term data available
Lembo A et al., 2016; RCT	636 IBS patients who responded to Rifaximin and relapsed	Rifaximin 550 mg three times daily for 2 weeks	Rifaximin has been shown to be effective in reducing abdominal pain, bloating, and loose stools, including patients who experienced symptom relapse after an initial course of therapy	No data available regarding repeated rifaximin administration
Fodor AA et al., 2019; RCT	103 IBS patients	Rifaximin 550 mg three times daily for 2 weeks	A transient effect on gut microbiota composition was observed, characterized by a reduction in the relative abundance of *Enterococcaceae*, *Peptostreptococcaceae*, and *Enterobacteriaceae*	Data were obtained exclusively from fecal samples using 16S rRNA gene sequencing
Diverticular disease				
Papi C et al., 1995; RCT	168 SUDD patients	Glucomannan 2 g/day + Rifaximin 400 mg two times daily for 7 days every month	After 12 months, a higher proportion of patients in the rifaximin group were symptom-free or only mildly symptomatic	No statistical difference in diarrhea severity
Latella G et al., 2003; RCT	968 SUDD patients	Glucomannan 4 g/day + Rifaximin 400 mg two times daily for 7 days every month	After 12 months, a higher proportion of patients in the rifaximin group experienced reductions in abdominal pain and diarrhea	Study design Absence of microbiological analysis
Colecchia A et al., 2007; RCT	307 SUDD patients	Rifaximin 400 mg two times daily for 7 days every month	Reductions were observed in lower abdominal pain, bloating, tenesmus, and abdominal tenderness	Study design Absence of microbiological analysis
De Vincentis A et al., 2021; RCT	43 SUDD patients	Rifaximin 800 mg/day for 7 day	Increased abundance of *Bacteroidaceae*, *Citrobacter*, Bifidobacteria, *Lactobacilli*, *Coprococcus*, and *Faecalibacterium prausnitzii*	Small sample size Patients not naïve to rifaximin

IBS, irritable bowel syndrome; RCT, randomized controlled trial; SUDD, symptomatic uncomplicated diverticular disease.

Due to its eubiotic and anti-inflammatory properties, rifaximin-α is also used for managing SUDD. Although it included only four trials, a meta-analysis by Bianchi et al.^146^ demonstrated the efficacy of rifaximin-α in alleviating SUDD symptoms. Patients treated with the antibiotic experienced significantly greater symptom relief compared to controls, with a number needed to treat (NNT) of 3 (*p* < 0.0001). In addition, the rifaximin group showed a slightly lower complication rate (−1.7%, *p* = 0.03).^146^ These findings were supported by a systematic review that analyzed 14 prospective clinical trials on SUDD. However, the review also indicated that rifaximin had limited efficacy in preventing acute diverticulitis, with an NNT of 57.^147^ In addition, a recent RCT found that rifaximin therapy significantly altered microbial diversity in SUDD patients. Post-treatment fecal samples showed an increased abundance of *Bacteroidaceae*, which exert anti-inflammatory activities, *Citrobacter*, Bifidobacteria, Lactobacilli, *Coprococcus*, and *F. prausnitzii*, alongside a reduction in some bacterial populations such as *Christensenellaceae*, often elevated in case of prior diverticulitis, *Eggerthella lenta*, another potential pathobiont, and *Ruminococcus* associated with bloating symptoms.^129,148^

Recently, rifaximin has been shown to be beneficial in both preventing and treating recurrent *C. difficile* infection. Major et al.^149^ demonstrated that administering rifaximin at a dose of 400 mg two to three times daily for a 4-week period effectively reduces the recurrence of *C. difficile*, regardless of the previous therapeutic regimen. Further evidence supporting the positive role of rifaximin in treating *C. difficile* infection comes from a meta-analysis of eight clinical trials, which confirmed that this nonabsorbable antibiotic is not only a potential treatment for mild to moderate *C. difficile* infection but may also serve as an adjunct therapy to prevent subsequent recurrences.^150^ However, these evidences still need to be confirmed in additional high-quality studies to be endorsed in international guidelines.^151^

### Fecal microbiota transplant

Fecal microbiota transplant (FMT) is an emerging therapeutic technique in which the gut microbiota from a healthy donor’s fecal sample is transferred to a recipient patient.^152^ Currently, the only clinically approved indication for FMT is the treatment of recurrent or persistent *C. difficile* infection.^153^ The rationale for its use is that *C. difficile* infection is a dysbiotic condition that can be reversed through microbiota restoration from a healthy donor. Given the high response rate of FMT treatment in *C. difficile* infections, its potential application in other dysbiosis-related conditions, such as IBS, has been explored.

Despite the numerous studies investigating FMT’s role in IBS treatment, there is insufficient evidence to support its routine clinical use (Table 2). As a result, most current guidelines advise against FMT for IBS patients outside of research settings.^37,124^

**Table 2.** Main studies reporting the efficacy of FMT in IBS.

Author and design	Study population	Intervention	Findings	Main limitations
IBS				
Johnsen PH et al., 2018; RCT	83 IBS patients	FMT administered via colonoscopy	FMT significantly reduced the IBS-SSS score in treated patients after 3 months	No information reported regarding single symptoms Not performed microbiota analysis
Halkjær SI et al., 2018; RCT	52 IBS patients	FMT administrated via capsules for 12 days	The placebo group exhibited a significant decrease in IBS-SSS scores at 3 and 6 months after FMT	All IBS subtypes were included Donor-mix technique for capsules preparation
Aroniadis OC, et al., 2019; RCT	48 IBS patients	FMT administrated via capsules for 3 days	Significant improvements in IBS-SSS, IBS-QoL, and BSFS were observed in both groups at 12 weeks of follow-up	No washout period Possible carryover effects
El-Salhy M et al., 2020; RCT	165 IBS patients	FMT administered via gastroscope	FMT was associated with significant reductions in abdominal pain, bloating, and fatigue at 3 months, as well as improvements in quality of life	Lack of data regarding long-term effects
Lahtinen P et al., 2020; RCT	49 IBS patients	FMT administered via colonoscopy	The FMT group exhibited a transient reduction in IBS-SSS	Small sample size No data regarding repeated infusions
Holvoet T, et al., 2021; RCT	64 IBS patients	FMT administered via nasojejunal probe	The FMT group demonstrated significant improvements in abdominal discomfort, stool frequency, and urgency	Small sample size Data obtained only from fecal samples sequencing 16S rRNA gene
El-Salhy M et al., 2022; RCT	125 IBS patients	FMT administered via gastroscope	A sustained significant reduction in IBS-SSS and improvement in IBS-QoL were observed in the FMT group at 3-year follow-up	Low response rate in the placebo group Limited microbiological analysis

BSFS, Bristol Stool Form Scale; FMT, fecal microbiota transplant; IBS, irritable bowel syndrome; IBS-QoL, quality of life; IBS-SSS, irritable bowel syndrome symptom severity scale; RCT, randomized controlled trial.

However, some studies suggest that FMT may have beneficial effects on IBS symptoms. Johnsen et al. conducted the first-ever RCT about the role of FMT in IBS treatment. They compared IBS patients who received a heterologous transplant (from a selected donor) with those who received an autologous transplant (their own microbiota). After 3 months, a significantly higher proportion of patients in the donor-transplant group achieved a meaningful reduction in IBS Severity Scoring System (IBS-SSS) compared to the autologous group (65% vs 43% of patients who achieved a significant response, *p* = 0.049). However, after 12 months, the difference between the two groups significantly diminished.^154^

Further support for the beneficial role of FMT in alleviating IBS symptoms comes from a larger RCT by El-Salhy et al. In this study, 165 patients were randomized into three groups: a placebo group (receiving autologous FMT), a 30 g FMT group, and a 60 g FMT group, with donor feces administered via gastroscopy. Three months post-transplant, the FMT groups showed a significantly higher response rate—defined as a reduction of at least 50 points in the IBS-SSS—compared to the placebo group (76.9% and 89.1% vs 23.6%, respectively; *p* < 0.0001). Notably, there was a significant difference in clinical response between the 30 and 60 g FMT groups (*p* < 0.0001). The improvement in IBS-SSS scores correlated with increased levels of *Lactobacillus* spp. and *Alistipes*.^155^ The same research group also conducted a 3-year follow-up of this population. The response rates at that time were 27.0% for the placebo, 64.9% for the 30 g FMT group (*p* < 0.1), and 71.8% for the 60 g FMT group (*p* < 0.001). Factors positively associated with a favorable clinical response included female sex, low baseline levels of *Lactobacillus* spp., severe IBS symptoms, and having the IBS-D or IBS-M subtype.^156^

On the other hand, several meta-analyses have yielded conflicting results regarding the efficacy of FMT in IBS treatment, primarily due to the heterogeneity of the included studies. Factors such as the number and the route of administrations, as well as donor selection, contribute to the variability in outcomes.^157,158^ Clearly, more high-quality studies are required to determine whether FMT could play a definitive role in IBS management.

Regarding SUDD, no studies have specifically evaluated the efficacy in symptomatic treatment. However, case reports have provided contradictory findings. Meyer et al.,^159^ described a case in which a patient with recurrent and multifocal episodes of acute diverticulitis underwent FMT for *C. difficile* infection and remained free of diverticulitis during a 20-month follow-up period. Conversely, another case reported the occurrence of acute diverticulitis shortly after FMT for *C. difficile* treatment.^160^ To determine whether FMT has a potential role in the management of diverticular disease, well-designed RCTs are essential.

## Conclusion

A substantial proportion of acute and chronic diarrhea conditions are associated with an imbalance in gut microbiota, highlighting the potential of microbiota-targeted therapies in improving disease management. Probiotics, antibiotics, and FMT have shown promising results in modulating the gut microbiota and alleviating symptoms. However, further research is essential to identify patient subgroups that are most likely to benefit from microbiota-directed therapies.

## Declarations

**Ethics approval and consent to participate:** Not applicable.

**Consent for publication:** Not applicable.

**Availability of data and materials:** Not applicable.
